# Role of agonistic autoantibodies against type-1 angiotensin II receptor in the pathogenesis of retinopathy in preeclampsia

**DOI:** 10.1038/srep29036

**Published:** 2016-07-06

**Authors:** Fang Liu, Yuxian Wang, Xiaofang Wang, Yanqian Zheng, Zhu Jin, Jianming Zhi

**Affiliations:** 1 Department of Ophthalmology, Shanghai Tenth People’s Hospital of Tongji University, 301 Yanchang Rd, Shanghai 200072, China; 2 Department of Obstetrics, The First Hospital, Shanxi Medical University, 85 South Jiefang Road, Taiyuan, Shanxi 030001, China; 3 Department of Physiology, School of Medicine, Shanghai Jiaotong University, 280 South Chongqing Road, Shanghai 200025, China

## Abstract

To investigate the mechanism underlying AT1-AA-induced retinopathy in severe preeclampsia by measuring the positive rate and titer of AT1-AA in plasma from women with severe preeclampsia and normal pregnant women to see whether AT1-AA titer was correlated with the grade of retinopathy. A preeclampsia rat model was also established by intravenous injection of AT1-AA extracted from the plasma of patient suffering from severe preeclampsia. The results showed that the plasma titer and positive rate of AT1-AA were significantly higher in women with severe preeclampsia than normal pregnant women. The antibody titer in cases of severe preeclampsia was associated with the grade of retinopathy, and positively correlated with the level of TNF-α and VEGF. The animal experiment results showed that the modeled rats presented symptoms very similar to symptoms of human preeclampsia, including retinopathy. Ocular fundus examination showed retinal microvascular abnormalities, hemorrhaging and leakage in the severe preeclampsia. Morphological changes included edema, thickening of the INL and ONL, and pigment atrophy. TNF-α and VEGF levels were increased in the vitreous humor and retina of the model rats. Our studies results suggest that abnormal expression of AT1-AA could induce damage to retinal capillary endothelial cells and increase vascular permeability, resulting in retinopathy.

Preeclampsia is a pregnancy-specific syndrome characterized by hypertension and associated proteinuria late in pregnancy of previously normotensive women. This condition result in life-threatening complications approximately 3–12% pregnancies. The etiology remains unknown, but an imbalance in circulating placental anti- angiogenic protein and growth factors is believed to contribute to the pathogenesis of preeclampsia^1^, involving vascular lesions and endothelial dysfunction of many important organs, including the eyes^2^.

Visual change is an important feature of preeclampsia. Visual disturbance is reportedly observed in 30–100% patients with severe preeclampsia^3^. severe preeclampsia is known to be associated with severe retinopathy similar to hypertensive retinopathy. Visual obscuration, scotoma, photopsia, cortical blindness, visual loss, retinal and vitreous hemorrhage are often observed in some preeclamptic patients. Ocular fundus examination can reveal a decreased retinal arterial-to-vein ratio, diffuse retinal edema, retinal hemorrhage and exudation, and serous retinal detachment^4^

The underlying mechanism responsible for the pathogenesis of preeclampsia remains unknown. In recent years, accumulative evidence indicates that immune abnormalities play a role in the pathogenesis of preeclampsia. Numerous studies^5^,^6^,^7^ have demonstrated that angiotensin II type I receptor agonistic autoantibody (AT1-AA) is an additional risk factor associated with the increased incidence of preeclampsia. By binding to and activates the AT1 receptor, AT1-AA exhibits an agonist-like activity similar to AT1 receptor. This stimulatory positive chronotropic effect is directly or indirectly involved in the pathogenesis of preeclampsia^8^.

Irani *et al*.^9^ reported that the effect of AT1-AA on pathophysiology of preeclampsia was mediated by a paracrine pathway by increasing synthesis and release of tumor necrosis factor (TNF-α) via AT1 receptor activation. Our previous study^10^ showed that TNF-α is a major mediator of AT1-AA induced cardiomyocyte apoptosis. TNF-α is a major cytokine that is up-regulated in various retinal vascular diseases such as diabetic retinopathy^11^, uveitis^12^ and rheumatoid corneal ulcerations^13^. TNF-α acts as a potent mediator of inflammation, angiogenesis and apoptosis, induces vascular endothelial growth factor (VEGF), and indirectly stimulates retinal neovascularization^14^. Lichtlen *et al*.^15^ reported that up-regulation of TNF-α led to the loss of microvascular cells in the retina.

VEGF is a potent angiogenic factor and vaso-permeability factor whose expression is increased by hypoxia. Ample evidence strongly indicates that VEGF is involved not only in ischemic retinal neovascularization but in early stages of retinopathy in preeclampsia^16^. AT1 can stimulate the release of VEGF from human vascular tissues. Increased expression of VEGF in the retina was observed in hypertensive patients and has been implicated in the pathogenesis of hypertensive retinopathy. Inhibition of AT1 including ACEI and AT1 receptor blockers and anti-VEGF was reported to reduce proliferative hypertensive retinopathy^17^,^18^.

The aim of the present study was to examine plasma AT1-AA titer in severe preeclampsia and determine whether change in AT1-AA level was associated with TNF-α and VEGF. To evaluate the *in vivo* pathophysiological consequences of AT1-AAs to the retina, we introduced AT1-AA purified from severe preeclampsia into pregnant rats on day 13 of gestation to determine the effect of AT1-AA on hypertensive retinopathy and level of TNF-α and VEGF in vitreous humor. Furthermore, we investigated whether AT1-AA-elicited pathological changes could stimulate TNF-α and VEGF production in the retina, and whether this effect could be blocked by AT1 receptor antagonists.

## Results

### Maternal clinical characteristics

A total of 87 women were included in the study, including 40 normal controls and 45 preeclamptic patients. The mean age of the two groups was 29.8 ± 6.4 (rang 21–45) and 29.1 ± 7.2 (rang 21–42) years, respectively. Systolic blood pressure (SBP), diastolic blood pressure (DBP) and urine protein in patients with severe preeclampsia were significantly higher than those in normal pregnant women. Clinical characteristics of the women in the two groups are shown in Table 1.

### Incidence of retinopathy in severe preeclampsia patients

**Figure 1:**
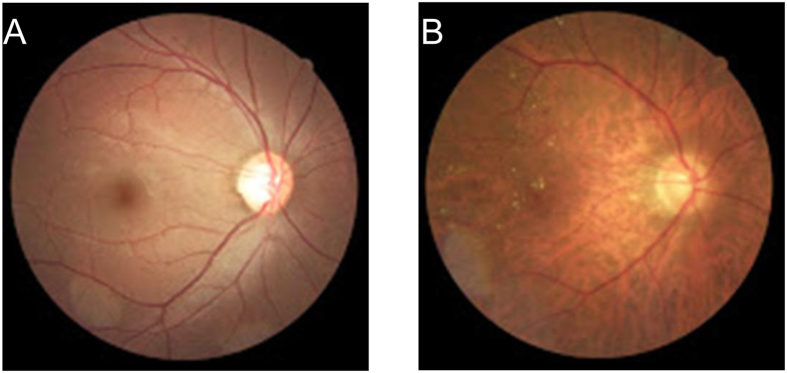
Color fundus photograph. (**A**) a normal pregnant women at 36 weeks of pregnancy, ocular fundus photography showed normal retinal structure; (**B**) a woman with severe preeclampsia 13 days after cesarean section, ocular fundus photography showed macular edema with striae and yellowish opaque retinal lesions.

The frequency of retinopathy in severe preeclampsia group was significantly higher than that in normal pregnancy group (31/45 *vs.* 2/40, p < 0.001). According to retinal vascular grading, Grade 1, 2, 3 and 4 retinal change was observed in 17 (37.8%) cases, 8 (17.8%) cases, 4 (8.9%) cases and 2 (4.4%) cases in severe preeclampsia group, respectivley (Table 2). Chi-square test showed a significant correlation between the ocular fundus change and BP (p < 0.05) (Table 3). Blurring vision and peripheral visual field loss were present in two patients of severe preeclampsia group (Fig. 1). In the normal pregnancy group, only 2 cases showed slight changes in the pigment epithelium.

### Clinical significance of AT1-AA titer in severe preeclampsia

**Figure 2:**
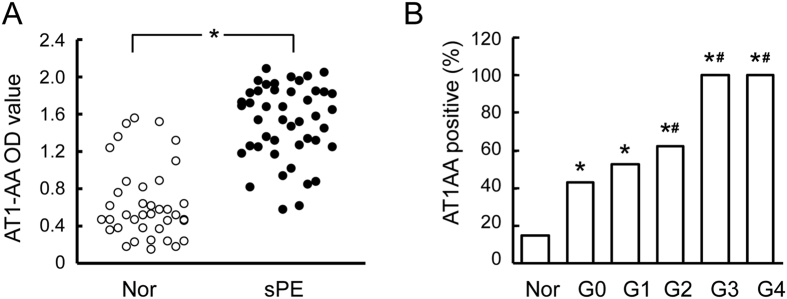
AT1-AA level in severe preeclampsia. Relative to the normal pregnancy group, AT1-AA titer was significantly increased in patients with severe preeclampsia (**A**). The number of AT1-AA positive patients was correlated with the grade of retinopathy (**B**). Values are mean ± SD. *p < 0.05 *vs.* normal pregnant group; ^#^P < 0.05 *vs.* grade 0, respectively.

There were 57.8% (26/45) AT1-AA serum positive cases in severe preeclampsia group *vs.* 15.0% (6/40) cases in normal pregnancy group (p < 0.001). The geometric mean titer of AT1-AA in severe preeclampsia group was significantly higher than that in normal pregnancy group (1:34.4 ± 6.8 *vs.* 1:136.2 ± 12.6, p < 0.001) (Fig. 2A). The correlation of plasma AT1-AA with retinopathy was analyzed in severe preeclampsia group. As shown in Fig. 1B, the number of AT1-AA positive patients increased with the grade of retinopathy: 52.94% (9/17) in Grade 1, 62.5% (5/8) in Grade 2, 100% (4/4) in Grade 3, and 100% (2/2) in Grade 4 (Fig. 2B). In addition, the AT1-AA titer was also significantly correlated with BP level: 39.1% (9/23) in low-BP group (SBP < 150 mmHg, DBP < 100 mmHg), and 61.5% in high-BP group (SBP > 150 mmHg, DBP > 100 mmHg) (Table 3).

### Correlation between plasma AT1-AA titer and VEGF and TNF-α level

**Figure 3:**
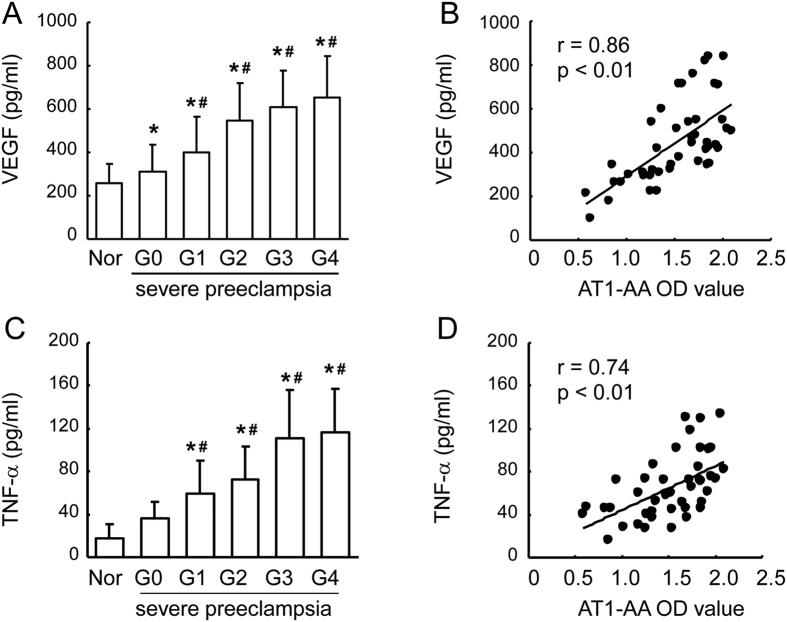
Plasma VEGF and TNF-α level in severe preeclampsia and normal pregnancy groups. Increased level of VEGF was detected in retinopathy grade (**A,C**). Scatter plots show a positive linear correlation between plasma VEGF and TNF-α level and AT1-AA titer in severe preeclampsia (**B,D**). Values are mean ± SD. *p < 0.05 *vs.* normal pregnancy group (Nor); ^#^P < 0.05 vs. grade 1, respectively.

Plasma TNF-α and VEGF levels in severe preeclampsia group were detected by ELISA. As shown in Fig. 3, plasma levels of TNF-α and VEGF in severe preeclampsia group were significantly increased as compared with normal pregnancy group (585.5 ± 38.5 pg/ml and 308.5 ± 141.3 pg/ml *vs.* 1.4 ± 17.5 pg/ml and 174.8 ± 61.5 pg/ml, p < 0.001 and p < 0.01), and these levels showed an increasing trend with the grade of retinopathy. Correlation analysis showed that there was a significant positive correlation between the plasma AT1-AA titer and plasma levels of VEGF and TNF-**α** (Fig. 3).

### Characteristics of pregnant rats

As shown in Table 4, there was no significant difference in initial SBP and body weight between the pregnant rats before treatment. However, SBP increased significantly in Group A 5 days after antibody injection as compared with Group N. There was an approximately two-fold increase in albuminuria in the Group A when compared with the pregnant Group N (P < 0.05). Various indexes including body weight, body length fetal rats were significantly smaller in Group A than those in Group N. However, these indexes did not undergo significant change in the fetal rats of Group L.

### Ocular fundus examination and fluorescence angiography

**Figure 4:**
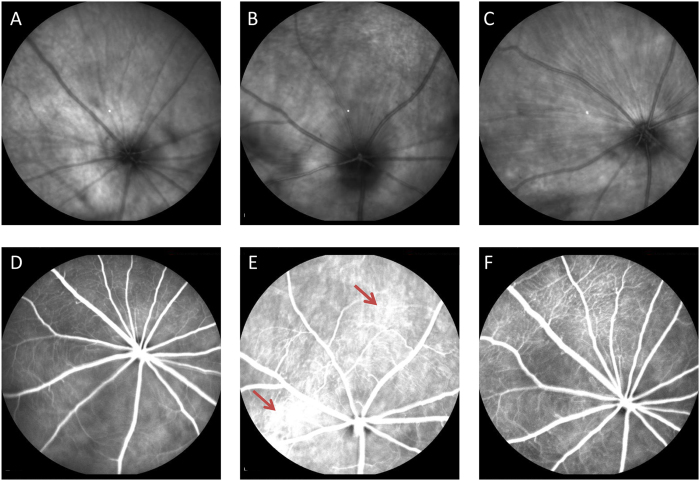
Fundus examination and fluorescein angiography in rat eyes. (**A,D**) Group N rat retinal fundus showing fine networks of blood vessels; (**B,E**) Group A rat fundus showing thin and irregular vascular networks with intensive light-reflection; (**C,F**) Group L rat showing improved morphology of retinal vascular network.

**Figure 5:**
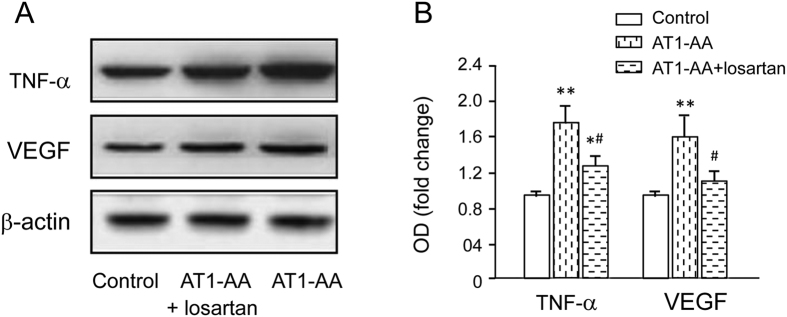
The effect of AT1-AA on retinal level of VEGF and TNF-α expression in the rat retina 10 days after injection. **(A**) Retinal levels of VEGF, TNF-α and β-actin were measured by Western blot. (**B**) The expression levels of VEGF and TNF-α were quantified by densitometry, normalized to β-actin levels, and expressed as percentages of the respective control values. Values are mean ± SD. *p < 0.05, **p < 0.01 *vs.* Group N; ^#^P < 0.05, ^##^P < 0.05 *vs.* Group A, respectively.

Ocular fundus examination showed no abnormality in both eyes in Group N (Fig. 4AD,). In contrast, retinal microvascular abnormalities were observed in both eyes in Group A, including thinning of the retinal arteries, widening of the retinal veins, and venous tortuosity and dilation. The blood vessels in some areas of the eye fundus in Group A were spasming, and the vascular lumen was totally occluded with no sign of blood flow in some regions of the blood vessel network (Fig. 4B). FFA examination showed that the retinal artery became significantly thinner and locally atresic, or even disappeared; instead, neovessels were observable. During the late phase of angiography, some fluorescence leakage areas were observed in Group A (Fig. 4E, arrow) (Fig. 5). However, the structure of most blood vessels in Group N was normal, and only a small part of the change (Fig. 4C,F).

The mean retinal blood vessels (arterioles and venules) is shown in Table 5. The mean retinal vessel diameter (arterioles and venules) in the Group A was significantly lower than that Group N (P < 0.05). Vessel diameter (arterioles and venules) in the Group L was significantly higher than in the Group A (P < 0.05), although it was lower than that Group N (P < 0.05).

### Effect of AT1-AA on VEGF and TNF-α levels in the vitreous humor and retina

Ten days after injection (the next day postpartum), the vitreous humor levels of both VEGF and TNF-α in Group A were significantly higher than Group N. Compared with the control A, there was significant difference with respect to VEGF and TNF-α in Group L (Table 6).

The expression of VEGF and TNF-α in the retina was assayed by Western blot. The results showed that the protein level of VEGF increased by almost 2 fold (Fig. 3A,B), and TNF-α in the retina increased by 2.5 fold after AT1-AA injection. However, losartan (an AT1 receptor antagonist) could to a large extent abolish AT1-AA-induced increase of VEGF and TNF-α in the retina (Fig. 5).

### Effect of AT1-AA on retinal damage

**Figure 6:**
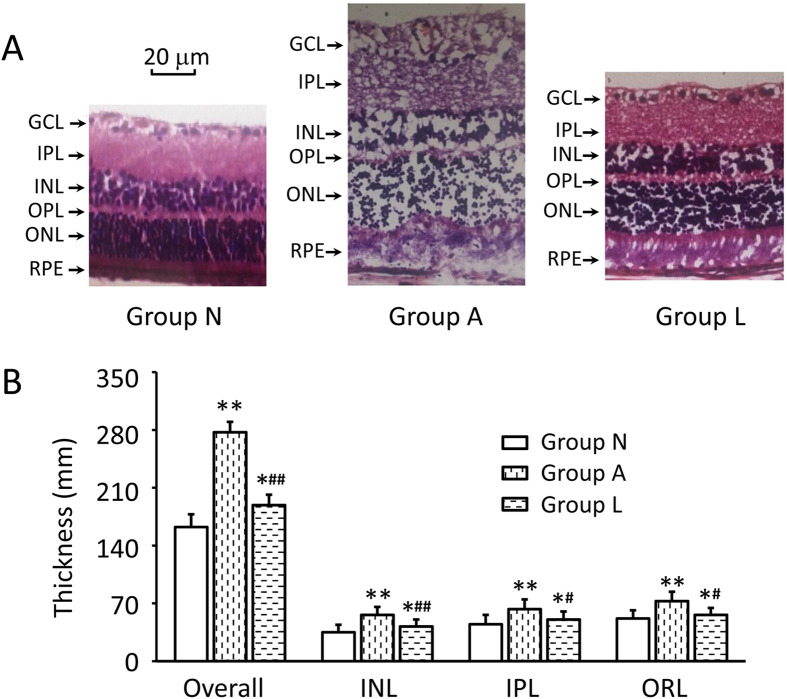
Histopathologic examination of the whole retina. **(A**) Representative photographs of rat retinas section from the three groups 10 days after injection. (**B**) Changes of the retinal thickness of each layer. In Group N, the GCL and INL were obvious and well organized. The INL in Group A was obviously thinner, and the number of GCL cells was significantly decreased, while in Group L, the retina was mildly edematous with a thicker INL as compared with Group N. GCL, ganglion cell layer; INL, inner nuclear layer; IPL, inner plexiform layer; OPL, outer plexiform layer; ONL, outer nuclear layer. Data are expressed as the mean ± SD (n = 6). *P < 0.05, **P < 0.01, vs. Group N; ^#^P < 0.05, ^##^P < 0.01, vs. Group A. Scale bar* *= 20 mm.

Ten days after AT1-AA injection, retinal morphological changes were evaluated. It was found that the retinas from Group N were normal in appearance. Compared with Group N, thickness of the retina in Group A was significantly increased, primarily attributable to significant degeneration of the cell bodies in the GCL and thinning of the INL, IPL, and ORL (Fig. 6A). The overall retinal thickness in Group A increased by 55.7% as compared with Group N (169.12 ± 15.23 *vs.* 263.56 ± 21.32 mm, p < 0.05) (Fig. 6B). Thickness of the INL, IPL and ORL increased by 47.6%, 65.4% and 58.9% respectively. GCL density was reduced by 68%. In contrast, losartan clearly protected against the retinal damage induced by AT1-AA, as represented by normal patterns of organization in the outer nuclear and plexiform layers. The thicknesses of the inner and outer retina and the GCL density in Group L were significantly smaller than those in Group A (P < 0.05), and significantly greater than those in Group N, confirming the protective effect of losartan (Fig. 6B).

### Transmission Electron Microscopy

**Figure 7:**
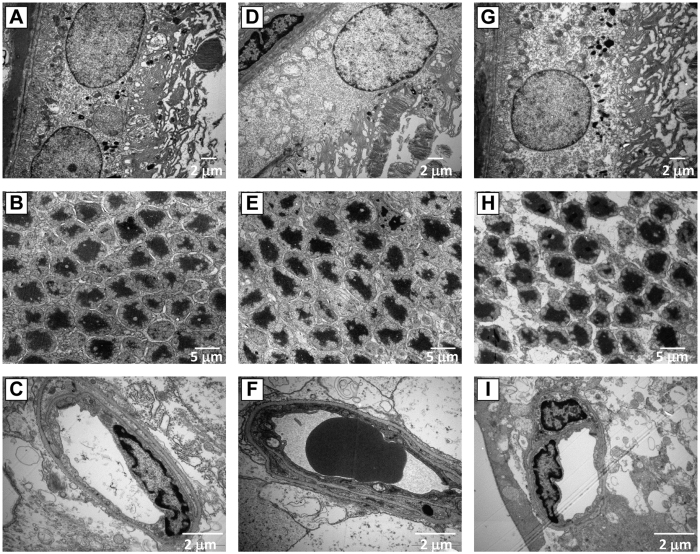
TEM analysis of the retina. Retinal morphology in Group N (**A–C**), Group A (**D–F**) and Group L (**G–I**). Disorganization of the villous structure of pigmented cytoplasm, cytoplasmic vacuolation and deterioration of cytoplasmic organelles were observed in rats treated with AT1-AA as compared with normal rats (**D**). Chromatin aggregation, and condensation, deformation and dissolution of photoreceptor cells were observed (**E**). The capillary lumen was narrow and capillary endothelial cells contained vacuoles (**F**). After treatment with losartan, retinal layers of the structure has improved significantly.

TEM examination showed that there was no significant abnormality in the ultrastructure of the rat retina in Group N (Fig. 7A–C), while many abnormalities were observed in Group A, including disorganization of the villous structure of pigmented cytoplasm, reduction of the pigmented epithelium with apparent nuclear damage, cytoplasmic vacuolation and deterioration of cytoplasmic organelles (Fig. 7D). In addition, the basement membrane was thickened; the endothelial cytoplasm contained many micropinocytic vesicles; the optic disc was edematous and the neurons were swollen. The thickness of inner nuclear layer and photoreceptor cell layer were decreased (Fig. 7E); retinal capillaries had pericytes with vacuoles and were slightly narrowed with irregular lumens; the retinal capillary basement membrane was significantly thickened; capillary endothelial cells contained vacuoles; and capillary pericytes were swollen and contained vacuoles (Fig. 7F). A certain degree of pathological change was observed in the ultrastructure of the retina in Group L compared with Group N, and degree of edema was significantly reduced as compared with the group A (Fig. 7G–I).

## Discussion

Studies in recent years have shown^19^,^20^ high-titer AT1-AA in the plasma of preeclamptic pregnant women, and this increased level of AT1-AA was reported to contribute to the pathophysiology of preeclampsia. In the present study, hypertensive retinopathy changes were seen in 68.9% of patients with severe preeclampsia, while hemorrhage, exudation and retinal detachment were observed in only a few patients in our series. At the same time, we demonstrated that positive AT1-AA was closely associated with severe preeclampsia. The frequency and titer of AT1-AA were significantly higher in patients with severe preeclampsia than those in normal pregnant women, and the grade of retinopathy was closely correlated with the titer of AT1-AA.

Based on the method reported in our previous study^11^, a preeclampsia animal model was established by injecting AT1-AA from the sera of preeclamptic rats via the tail vein. Five days after AT1-AA injection, SBP was significantly higher compared with Group N, and proteinuria appeared. The body weight, heart weight, body length and placental weight of the fetal rats were significantly lower or smaller in the AT1-AA group. These results suggest that the model was established successfully. Ocular fundus photography and fundus fluorescein angiography of the pregnant rats showed retinal hemorrhage, edema, telangiectasia and thickening in the fundus of the modeled rats 10 days after injection. Most of these lesions were attributed to vascular damage caused by AT1-AA.

Losartan, an AT1 receptor blocker hase been widely used to blood pressure- lowering, renal protection and cardioprotection in patients, but also has protective effects on retinal blood vessels in various eye diseases. Quigley *et al*.^21^ reported that losartan treatment decreases the retinal ganglion cell death caused by experimental intraocular pressure elevation in mice. Silva *et al*.^22^ provides evidence of the losartan in ameliorating diabetic retinal neurodegeneration, mitochondrial function, and oxidative balance. Our results show that 10 mg of losartan once daily can serve as an effective prevention and treatment retinopathy in preeclampsia rat model for induced by AT1-AA.

TNF-α is known to play a major role in various retinal vascular diseases through a range of pathogenic pathways such as endothelial and retinal cell injury, apoptosis, angiogenesis and vascular leakage. Injection of TNF-α into animal eyes induced breakdown of the blood-retina barrier^23^. Up-regulation of TNF-α led to the loss of microvascular cells in the retina, and its inhibition by TNF-α receptor molecules/ antibodies protected the retinal microvasculature^24^. Zhou *et al*.^8^ reported that injection of AT1-AA triggered the onset of preeclampsia and increased TNF-α production, placental apoptosis and fetal anomalies in pregnant mice. Our previous study^10^ showed that AT1-AA could induce the apoptosis of cultured cardiomyocytes by releasing TNF-α from cardiomyocytes, and blockade of these features could be diminished by AT1 receptor blocker losartan and soluble TNF-α inhibitor etanercept. To the best of our knowledge, the present study is the first to show that tail vein injection of AT1-AA into pregnant rats could increase TNF-α levels in the vitreous humor. So we thought that TNF-α could be one of the reasons why retinal vascular lesions were induced by AT1-AA in pregnant rats.

VEGF is another major cytokine involved in the development of retinal vascular diseases, and also a potent mediator of vascular remodeling and angiogenesis. Inhibition of VEGF production is associated with suppression of retinal neovascularization. Therefore, VEGF is implicated in the pathogenesis of retinopathy. Apart from hypoxia as the major stimulus for VEGF expression in the retina, angiotensin II is also a known stimulus for VEGF expression. Inhibition of the production of AT1 with angiotensin- converting enzyme (ACE) inhibition was reported to be associated with suppression of VEGF expression in experimental diabetes^25^. All components of the RAS, including AT1 receptors and AT2 receptors, have been demonstrated to be present in the retina. Previous studies have shown that increased VEGF expression induced by AT1 stimulation was mediated by the AT1 receptor. Nakamura *et al*.^26^ found that candesartan (an AT1 receptor antagonist) inhibited retinal pathological neovascularization, at least in part by suppressing the expression of VEGF receptor-2.

Liu and his colleagues^27^ found that a higher plasma AT1-AA titer may be associated with advanced progression of epithelial ovarian cancer and play an important role in the development progression of epithelial ovarian cancer by promoting cancer cell migration and angiogenesis. Our experimental results showed that tail vein injection of AT1-AA increased the VEGF level in the vitreous humor of pregnant rats, and treatment with losartan effectively reduced the vitreous humor level of VEGF. We therefore speculate that AT1-AA may increase the expression of VEGF in the retina through direct and indirect two ways. Like angiotensin II, AT1-AA can stimulate endothelial cells to produce VEGF, thus suppressing the expression of VEGF receptor by AT1 receptor activation. On the other hand, plasma AT1-AA is a potent vasoconstrictor that can increase intracellular Ca^2+^, which causes the smooth muscle to contract, thus reducing choroidal blood flow, resulting in retinal ischemia. Our recent study^28^ found that AT1-AA was able to cause amplification response to AT1 at physiological concentrations probably via the calcium-independent protein kinase C pathway, resulting in more severe retinal ischemia and hypoxia. Hypoxic exposure led to a significant increase in VEGF expression in both retinal capillary endothelial cells and Muller cells, thereby causing increased VEGF levels in vitreous humor. VEGF promotes endothelial cell differentiation, proliferation and migration by activating its tyrosine kinase receptors VEGFR1 and VEGFR 2, changing the extracellular matrix and breaking down the inner endothelial blood-retinal barrier, eventually causing endothelial cell injury, angiogenesis, retinal vascular permeability, retinal edema, visual impairment, and complete blindness in severe cases^29^.

It has been reported that increased VEGF expression in the retina has been reported in preeclampsia, and VEGF may play an important role in the early phases of pregnancy- associated angiogenic retinal diseases, thus the use of anti-VEGF agents has proven helpful^30^. Many studies^31^,^32^ have reported that plasma VEGF levels were significantly decreased in preeclampsia than those in normal pregnant women, and this may be related to higher levels of plasma soluble VEGF receptor 1 (sFlt-1).

In summary, the present study showed that the antibody titer of AT1-AA plasma and the incidence of retinal disease were significantly higher in women with severe preeclampsia than those in normal pregnant women, and that increased plasma VEGF level was strongly correlated with the grade of retinopathy. The antibody injection model of preeclampsia with retinopathy described here provides strong experimental support to our working hypothesis that AT1-AA causes retinopathy in severe preeclampsia. VEGF and TNF-α are involved in AT1-AA-mediated retinopathy. The biological properties of AT1-AA could be blocked by AT1 receptor blockers.

## Materials and Methods

### Human subjects

Enrolled in this study were 45 patients with severe preeclampsia who were hospitalized in the department of obstetrics and gynecology of our hospital between June 2014 and June 2015. The study was approved by the Ethics Committee of Shanghai Jiao Tong University School of Medicine, and written informed consent was obtained from all patients at study entry. All the experiments were carried out in accordance with the approved guidelines and regulations. All patients who fulfilled the diagnostic criteria of pregnancy induced hypertension (> 24 weeks of pregnancy, high arterial blood pressure and proteinuria). Simultaneously randomly selected 40 age-matched pregnant women were used as control. Exclusion criteria included patients with hypertension, diabetes mellitus, vasculitis, renal disease and retinal disease.

Ocular fundus examination was performed by ophthalmologists with a direct ophthalmoscope. Hypertensive retinopathy changes seen in right or left or both eyes represented positive findings in that patient. According to Keith Wagener classification, retinal changes were classified as: Grade 1, mild generalized arterial attenuation, particularly of small branches; Grade 2, more severe grade 1+ focal arteriolar attenuation; Grade 3, grade 2+ haemorrhages, hard exudates, cotton wool spots; and Grade 4, grade 3+ optic disc swelling (papilloedema).

### ELISA assay and antibody affinity purification

Peripheral venous blood samples were collected in sterile pre-cooled tubes containing EDTA and centrifuged at 2000 rpm for 20 min. The supernatant was collected for ELISA determination of AT1-AA and antibody purification. Plasma antibody titer was measured by using the enzyme-linked immunosorbent assay (ELISA) described previously^33^. AT1-AA was purified by MAb Trap Kit (Amersham, Piscataway, NJ, USA) according to the manufacturer’s instructions. Before use, the purified antibody was diluted with phosphate- buffered saline (3.2. mM Na_2_HPO_4_, 0.5 mM KH_2_PO_4_, 1.3 mM KCl, 135 mM NaCl, pH 7.4) to an antibody titer of greater than 1:160 by ELISA detected. VEGF and TNF-α were both measured using sandwich ELISA (R&D Systems, Inc., Minneapolis, MN, USA) in duplicate according to manufacturer’s instructions.

### Introduction of antibody into rats

Wistar pregnant rats were fed normal rat chow and tap water ad libitum with a 12:12 h light–dark cycle (lights on at 07:00 h, lights off at 19:00 h) at a constant ambient temperature (23 + 2°C) and humidity (60% + 5%). All of the experimental protocols were approved by The Institutional Animal Care and Use Committee of Shanghai Jiaotong University. All of the methods were carried out in accordance with the Guide for the Care and Use of Laboratory Animals.

The pregnant rats were randomized into saline control group (Group N, *n *= 6), AT1-AA treated group (Group A, *n* = 6), and AT1-AA + losartan treated group (Group L, *n* = 6). In Group A, AT1-AA (100 μL PBS, titer >1:640) was administered to the pregnant rats via tail vein injection at day 13 and again at day 14 (term = 22–23 days). Some rats also received losartan at a dose of 10 mg/kg/day starting from day 1 after injury to the endpoint of the study as Group L. Systolic blood pressure (SBP) of the pregnant rats was measured by a tail-cuff system (AD Instruments) at daily intervals after AT1-AA treatment. Urine was collected in metabolic cages for 12 h at gestation day 18 for measurement of total albumin.

### Ocular fundus examination and fluorescence angiography

On the second day after delivery, the rats were anesthetized with sodium pentobarbital (50 mg/kg intraperitoneally), the pupils were dilated with 1% tropicamide, and the cornea was kept moist using 1% carboxymethylcellulose sodium eye drops. The rats were injected intraperitoneally with 10% fluorescein sodium (Alcon, USA) at a dose of 1.5 ml/kg. Images were recorded 3 to 240 seconds after injection. Fundus fluorescein angiography (FFA) was performed with a digital fundus camera (TRC-50EX; Topcon, Tokyo, Japan). Final fundus photographs were used for estimating the arteriolar and venular diameter. The diameter of retinal vessels was estimated by the methodology of Gupta *et al*.^34^. Diameter measurements were obtained at three different locations along each vessel near optic disk, with an average of three measurements.

### Histological observation and morphological change

After ocular fundus examination, both eyeballs were enucleated. Vitreous humor samples obtained from both eyes were used to determine VEGF and TNF-a levels by ELISA. The left retina was fixed in 4% paraformaldehyde at 4°C for 24 h. After fixation, the anterior segment was removed, and the posterior eyeball was dehydrated in a graded ethanol series and embedded in paraffin. For hematoxylin and eosin (HE) staining, samples were cut into 5-μm-thick retinal cross sections and observed under a light microscope (Leica, Heidelberg, Germany).

To quantify the AT1-AA for damage to the retina, we measured different layer thicknesses to quantify the degree of cell loss. The overall retinal thicknesses (from the inner limiting membrane to the pigment epithelium), the outer retinal layers (ORL, consisting of the outer nuclear and outer plexiform layers), the inner nuclear layer (INL), and the inner plexiform layer (IPL) were measured. The retinal layers measured are indicated in Fig. 6A. The number of cells in the ganglion cell layer (GCL) was calculated using the linear cell density (cells per 200 μm), and 3 sections per eye were averaged.

The right retina was obtained and a peripheral area of 1 mm^2^ was cut and fixed in 2.5% glutaraldehyde for observation of the ultramicrostructure of the retina under a Philips CM120 transmission electron microscope (TEM, Philips Electronics Ltd., Amsterdam, Netherlands). Most of the remaining right retina was used for determination of VEGF and TNF-a levels by Western blot.

### Western blot analysis of VEGF and PEDF

Western blot analysis was performed as described previously^35^. Briefly, the retina was homogenized by sonication at 4 °C, and the protein concentration of the supernatant was measured with a protein assay reagent kit (Bio-Rad Laboratories). Soluble protein (80 μg) from each sample was separated by 10% SDS-PAGE and then transferred to PVDF membranes (Millipore) using standard electroblotting procedures. The blots were then blocked and incubated overnight at 4 °C with anti-TNF-α (Abcam, Cambridge, MA, USA), anti-VEGF (Abcam) primary antibodies. Immunolabeling was detected using an enhanced chemiluminescence kit (GE Healthcare, Pittsburgh, PA, USA) according to the manufacturer’s instructions.

### Statistical analysis

All data were calculated as mean ± SD. Positivity was defined as a ratio of (sample A - blank A)/(negative control A - blank A) ≥2.1. Antibody titer was reported as geometric mean. The positive rates in the two groups were compared with chi-square test. One-way ANOVA test was used to determine significant differences between groups. The t-test was applied for comparing two independent sample means, and the one-way ANOVA was used for comparing means of more than two samples. Data were analyzed using SPSS 16.0 (SPSS, Chicago, Illinois, USA). P < 0.05 was considered statistically significant.

## Additional Information

**How to cite this article**: Liu, F. *et al*. Role of agonistic autoantibodies against type-1 angiotensin II receptor in the pathogenesis of retinopathy in preeclampsia. *Sci. Rep.* **6**, 29036; doi: 10.1038/srep29036 (2016).

## Figures and Tables

**Table 1:** Clinical characteristics of normal and preeclamptic women included in this study (mean ± SD).

Parameter	Normal pregnancy (n = 40)	Severe preeclampsia (n = 45)
Age (years)	29.8 ± 6.4	29.1 ± 7.2
SBP (mmHg)	118.2 ± 7.4	148.7 ± 15.7*
DBP (mmHg)	75.2 ± 5.9	106.9 ± 11.5*
Urinary protein (mg/24 h)	NA	2.05 ± 0.67

^*^P < 0.05 vs normal pregnant.

**Table 2:** Retinal changes in normal and preeclamptic women included in this study.

Grades of retinopathy	Severe preeclampsia (*n* = 45)		Normal pregnancy (*n* = 40)	
	Number of changes	Percentage (%)	Number of changes	Percentage (%)
No changes	14	31.1	36	90
Grade 1	17	37.8	2	5.0
Grade 2	8	17.8	0	0
Grade 3	4	8.9	0	0
Grade 4	2	4.4	0	0

**Table 3:** Correlations between retinopathy and different variables in severe preeclampsia group.

Parameter	Retinal Change (Grade)					Total	P
	G0	G1	G2	G3	G4		
Age (years)							
20~30	7	8	4	2	1	22	0.46
30~40	6	8	3	2	1	20	
>40	1	1	1	0	0	3	
Blood pressure (mmHg)							
SBP<150, DBP<100	8	7	3	1	1	20	0.001
SBP>150, DBP>100	6	10	5	3	1	25	
AT1-AA titer							
<1:40	8	8	3	0	0	19	0.014
>1:40	3	2	2	0	0	7	
>1:160	2	4	1	2	1	10	
>1:640	1	3	2	2	1	9	

**Table 4:** Maternal and fetal characteristics.

Parameter	n	Group N	n	Group A	n	Group L
		mean ± SD		mean ± SD		mean ± SD
Initial mother weight (g)	6	217.8 ± 12.2	6	220.1 ± 12.2	6	219.8 ± 12.7
Mother weight at term(g, day 21 of pregnancy)	6	393.7 ± 19.3	6	368.4 ± 18.2*	6	389.9 ± 20.5#
Initial mother SBP (mmHg)	6	109.8 ± 8.6	6	108.6 ± 6.4	6	110.8 ± 7.2
Mother SBP at term (mmHg, day 21 of pregnancy)	6	112.7 ± 9.2	6	141.4 ± 11.5*	6	121.2 ± 10.9#
Albuminuria (mg/ 24 h, day 18 of pregnancy)	6	4.26 ± 1.05	6	11.35 ± 4.54*	6	6.27 ± 1.68#
Fetal body length (mm)	77	36.2 ± 1.7	75	34.7 ± 1.7*	74	36.0 ± 1.5#
Fetal prenatal body weight (g)	77	4.85 ± 0.18	75	4.32 ± 0.15*	74	4.78 ± 0.17#

^*^P < 0.05 vs Group N;

^#^P < 0.05 vs Group A.

**Table 5:** The effect of AT1-AA on retinal blood vessels (arterioles and venules) in the rat 10 days after injection (n = 6).

Group	Arterioles (μm)	Venules (μm)
Group N	58.92 ± 7.61	76.28 ± 9.21
Group A	36.87 ± 6.82**	49.48 ± 8.53**
Group L	49.12 ± 6.55^#^*	65.21 ± 8.98#

^*^P < 0.05,

^**^P < 0.01 *vs.* Group N;

^#^P < 0.05 *vs.* Group A.

**Table 6:** The effect of AT1-AA on vitreous humor levels of VEGF and TNF-α expression in the rat retina 10 days after injection (*n *= 6).

Group	VEGF (pg/ml)	TNF-α (pg/ml)
Group N	142.38 ± 41.54	28.22 ± 8.42
Group A	235.54 ± 42.35**	42.81 ± 9.37**
Group L	187.62 ± 52.58#*	33.08 ± 8.64#

^*^P < 0.05,

^**^P < 0.01 *vs.* Group N;

^#^P < 0.05 *vs.* Group A.
